# Telerehabilitation Intervention in Patients with COVID-19 after Hospital Discharge to Improve Functional Capacity and Quality of Life. Study Protocol for a Multicenter Randomized Clinical Trial

**DOI:** 10.3390/ijerph18062924

**Published:** 2021-03-12

**Authors:** José-Manuel Pastora-Bernal, María-José Estebanez-Pérez, Guadalupe Molina-Torres, Francisco-José García-López, Raquel Sobrino-Sánchez, Rocío Martín-Valero

**Affiliations:** 1 Department of Physiotherapy, Faculty of Health Science, University of Granada, 18071 Granada, Spain; 2 Department of Physiotherapy, Faculty of Health Science, University of Malaga, 29071 Málaga, Spain; mariajoseestebanezperez@gmail.com (M.-J.E.-P.); rovalemas@uma.es (R.M.-V.); 3 Department of Nursing Science, Physiotherapy and Medicine, University of Almeria, 04120 Almeria, Spain; guada.lupe@ual.es; 4 Department of Physiotherapy, University of Osuna, 41640 Seville, Spain; raqsobsan@gmail.com

**Keywords:** COVID-19, telerehabilitation, physiotherapy, discharge from hospital, confinement, COVID-19 sequelae

## Abstract

COVID-19 can cause important sequels in the respiratory system and frequently presents loss of strength, dyspnea, polyneuropathies and multi-organic affectation. Physiotherapy interventions acquire a fundamental role in the recovery of the functions and the quality of life. Regarding the recovery phases after hospital discharge, the current evidence available is very preliminary. Telerehabilitation is presented as a promising complementary treatment method to standard physiotherapy. The main objective of this research is to evaluate the effectiveness of a personalized telerehabilitation intervention after discharge from hospital for the improvement of functional capacity and quality of life compared to a program of health education and/or care in a rehabilitation center. As secondary objectives, to identify the satisfaction and perception of patients with the telerehabilitation intervention and the presence of barriers to its implementation, as well as to evaluate the cost-effectiveness from the perspective of the health system. This study protocol will be carried out through a single blind multicenter randomized clinical trial in the south of Spain. We hypothesize that the implementation of a telerehabilitation program presents results not inferior to those obtained with the current standard intervention. If the hypothesis is confirmed, it would be an opportunity to define new policies and interventions to address this disease and its consequences. Trial registration NCT04742946.

## 1. Introduction

Severe acute respiratory syndrome coronavirus 2 (SARS-CoV-2) causes coronavirus disease 2019 (COVID-19). On 11 March 2020, COVID-19 was categorized as a pandemic and its outbreak was declared a public health emergency of international concern [1,2,3]. Current reports estimate that 80% of cases are asymptomatic or mild; 15% of cases are severe (infection requiring oxygen supply); and 5% are critical patients requiring ventilation and life support [2,4,5,6]. The average time from symptom onset to recovery is two weeks when the illness is mild; and three to six weeks when it is severe or critical. It should be noted that between 75–80% of hospitalized patients will be admitted to hospital for a long period of time (±21 days) [7].

COVID-19 can cause important sequelae in the patient after the virus has been overcome, one of the main ones being respiratory system involvement by bilateral pneumonia. Loss of strength, dyspnea, polyneuropathy and multiorgan involvement (liver, myocarditis and even brain damage) are also common [5,8,9]. Consequently, physiotherapy is playing a vital role in this pandemic and will be fundamental in improving the health of many of the patients. Recent studies already indicated a loss of pulmonary capacity of between 20% and 30% [10]. Therefore, it is a multiorgan disease with a wide and heterogeneous range of sequelae, the extent of which is not yet known [11].

### 1.1. Physiotherapy in Patients with COVID-19

Physiotherapy has a very important role in the functional recovery of patients with COVID-19, both at the respiratory and motor levels in their different phases of evolution [12]. International studies and expert consensus begin to draw the roadmap for physiotherapists in the different stages of coronavirus both for patients in intensive care units and during their hospital stay on the ward [13], defining the available evidence on each modality of intervention [14]. An international group of experts in cardiorespiratory physiotherapy met to urgently develop a clinical practice guideline in the approach of physiotherapy to COVID-19. The group focused mainly on case management interventions in the acute hospital phase where rehabilitation intervention is essential to limit severity and promote early functional recovery [14]. Research has pointed out the indispensability of providing physiotherapy services including weekends and holidays [15].

With respect to the recovery phases, discharge from hospital and subsequent confinement for up to 14 days, according to health recommendations, the data currently available are preliminary [16]. In this recovery and discharge phase, two types of patients are differentiated:

For patients with a mild-moderate respiratory process, the short-term goal will be to gradually restore physical and psychological condition with a series of aerobic exercise guidelines to restore pre-hospital exercise capacity [16].

For patients with a severe/critical process, the evidence indicates that at the time of discharge patients with COVID-19 may experience physical deconditioning, dyspnea secondary to exercise and muscular atrophy among other sequelae [17,18]. In these patients with a severe/critical process, the main physiotherapy interventions will be based on: patient education, aerobic exercise, strength and training exercises, secretion drainage and ventilatory techniques, if the patient’s clinical manifestations so require, exercises aimed at strengthening the peripheral muscles, changes in position and remaining active whenever possible and when the situation of clinical stability permits [17,18].

### 1.2. Telerehabilitation

This term is used to describe the provision of rehabilitation services at a distance, using communication technologies [19]. An expanded definition includes applications such as consultation, home care, monitoring, therapy and patient self-care that are provided in various locations including the home, community, health services and the workplace [20,21].

In addition to traditional physical therapy, studies based on telerehabilitation programs have published results of effectiveness, validity, non-interference and important advantages in some neurological, cognitive, musculoskeletal and respiratory disorders, providing an opportunity to define new policies and social interventions [22]. There is a growing interest in the use of technology for respiratory health interventions [23]. Telerehabilitation offers an alternative approach that can better meet the needs of patients with airway and lung impairments especially in terms of ease of access and elimination of travel [24] and in the case of COVID-19 by limiting their possible transmission by avoiding human-to-human contact.

Currently, health systems are immersed in a continuous process of innovation to improve the effectiveness of health services [25,26]. Telerehabilitation is presented as a promising complementary treatment method to standard physiotherapy, providing physiotherapy support at any time and in any place, both in the health field, community environment, and the home in several pathologies [27,28]. It has also been stated that the high viability and acceptability of this technology can be satisfactory to obtain an improvement in the quality of life of patients [29].

Although it is still too early to state with a high level of evidence the consequences of COVID-19 on functional capacity, the need to implement pulmonary rehabilitation programs in these patients is based in evidences in similar pathophysiology [30,31,32].

The main objective of this research is to evaluate the effectiveness of a personalized telerehabilitation intervention in patients diagnosed with COVID-19 after discharge from hospital for the improvement of functional capacity and quality of life compared to a program of health education and/or care in a rehabilitation center. As secondary objectives, to identify the satisfaction and perception of patients with the telerehabilitation intervention and the presence of barriers to its implementation, as well as to evaluate the cost-effectiveness from the perspective of the health system. The hypothesis of this research is that the implementation of a telerehabilitation (TR) program in patients with COVID-19 presents no inferior results in the improvement of functional capacity and quality of life compared to those obtained with a health education program and/or care in a rehabilitation center.

## 2. Material and Methods

**Figure 1:**
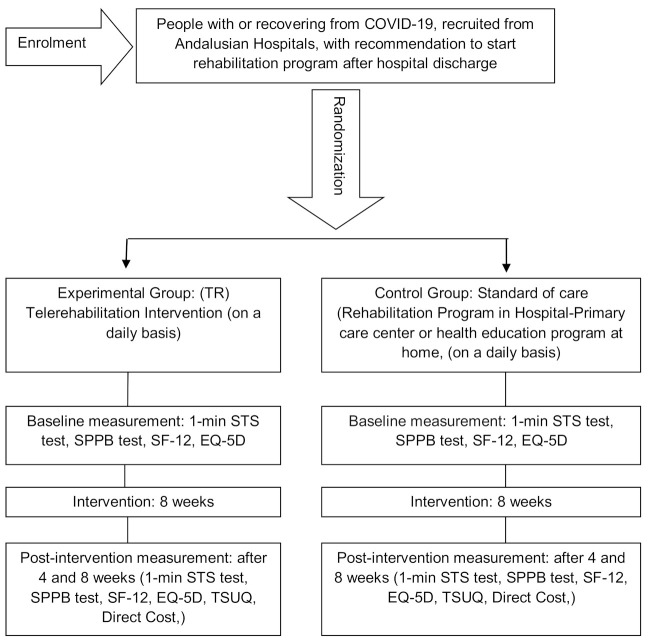
Study design.

This research is carried out by means of a single blind multicenter randomized clinical trial in patients residing in Andalucía (south of Spain) with a COVID-19 diagnosis who have been discharged from hospital. This research uses the guidelines on Standards for Quality Improvement and Excellence in Reporting (SQUIRE) [33] and will be carried out in accordance with CONSORT (Consolidated Standards of Reporting Trials) criteria [34]. A Standard Protocol Items Recommendations for Interventional Trials (SPIRIT) checklist is provided as Additional file 1, and the flow diagram for the study protocols is included as Figure 1.

### 2.1. Patients

The study includes adults diagnosed with COVID-19 with mild-moderate and severe-critical respiratory processes who have been treated in hospitals in (south of Spain) and who have been discharged from hospital. The fundamental ethical precepts according to the Helsinki Declaration [35] and Law 14/2007 of 3 July on Biomedical Research will be respected, guaranteeing the protection and confidentiality of data. This trial has the approval of the Andalucía Ethics Committee with HIP version 281020. Patients in the study are required to read and approve the consent form.

Patients must reside or stay in the Andalucía (south of Spain) during the intervention phase, have computer technology with an Internet connection at home (including one of the following devices: desktop personal computer, laptop, tablet or smartphone), and be able to access e-mail or WhatsApp frequently and reliably. Patients who are not in full cognitive capacity to use new technology tools will be excluded.

For the development of this research, an intentional non-probabilistic type of sampling will be used for the convenience of the study, due to the characteristics of the subjects.

A sample size of (*n* = 237) with a proportional distribution for each arm of the study (group TR = 118 + control group = 117) with an expected proportion of losses (R = 15%) and a confidence index IC = 95% was used.

Patients are recruited from hospitals rehabilitation and physiotherapy departments. Strategies to achieve an adequate inclusion of participants that reach the target size of the sample include the multidisciplinary collaboration of south of Spain hospitals, heads of rehabilitation services and Physiotherapist teams. The collaborators will be informed about the characteristics of the study in personal interviews and presentation of the project. Patient recruitment seeks to ensure socio-demographic diversity in relation to social origin, gender, ethnicity and education adapted to the particularities of the reference population in Andalucía and prior information on compliance with data protection laws. We will try to make sure that the sample data are homogeneous at the beginning of the study, trying not to have significant differences in demographic, medical and other variables.

### 2.2. Randomization and Single Blinding

Before patient inclusion, a research secretary will generate the allocation sequence and randomly assign patients consecutively with opaque sealed numbered envelopes. A computerized random number generator will be used. The research secretary will be instructed not to reveal the allocation sequence to any of the research team. The nature of the intervention in both groups does not allow blinding of patients and physiotherapists. It is therefore a single-blind study, where the evaluator does not know the nature of the intervention.

Study subjects are randomly assigned to either the TR group or the control group through a computerized randomization system. Subjects receive an initial evaluation based on clinical parameters and follow-up discharge reports. Data will be collected by an evaluator and integrated into research databases.

### 2.3. Intervention

The TR group receives a personalized program for 8 weeks, including at least one session per day and is done through a web and mobile application. The telerehabilitation application allows health professionals to create personalized exercise programs, hold video conferences with patients, generate videos, images and parameters of each exercise, as well as send them by email and follow up patients through the mobile application.

The TR program describes the exercises to be performed, the number of series and repetitions, and the criteria for progression, which will be based on the clinical guidelines published for patients with COVID-19 and described in the introduction section. The Physiotec software was selected by the research team to be best suited to the needs of the patients.

Patients are initially supervised by the research team who will conduct training sessions to ensure proper execution of the exercises and encourage patient adherence. Patients will be instructed to perform self-training by following the video exercises through the TR program, as well as a support document called COVID-19 Patient Telerehabilitation Manual. The number of synchronous sessions (1 to 1) via videoconference will be determined by the initial evaluation, limiting the interventions to 1 session per day of maximum 45–50 min, always adapted to the previous evaluation and to the needs of each patient.

Patients will be advised to refrain from any other specific training during the intervention period. Any deviation from the adherence and practice of the TR program is recorded on a daily basis, noting any adverse incidents.

Telerehabilitation and physical therapy interventions may include aerobic training—generate exercise patterns such as walking, fast walking, jogging, swimming, etc., starting at a low intensity and duration and increasing gradually; 20–30 min of session duration is recommended, 3–5 sessions/week, although it will always depend on the sensation of fatigue and/or dyspnea that each patient presents [18]. Progressive strength training is recommended working 1–3 muscle groups with a load of 8–12 repetitions, with 2-min training intervals. The frequency would be 2–3 sessions/week for a minimum period of 6 weeks, increasing the load by 5–10%/week [18]. Secretion drainage or ventilatory techniques to re-educate the respiratory pattern, improve ventilation, mobilize the thorax and favor secretion drainage, especially in those patients with chronic pathology prior to COVID-19 or who have a reduced pulmonary capacity to cause the disease [36].

Due to the lack of detailed knowledge of sequelae, a correct evaluation of each particular case is recommended before applying physiotherapeutic techniques [16]. The software and the mobile application for such intervention are provided and financed by exclusive license by the principal researcher. Customized exercise program examples are available at main author request.

The control group (CG) receives the standard of care recommended for patients discharged from hospital. Based on the individual needs of each subject, it consists of a rehabilitation program in their hospital or primary care center of reference, in the event that they present evidence of immunity and the evaluation of the sequelae requires it. If you do not present proof of immunity or if you test positive for COVID-19, you will receive a recommendation for confinement for a minimum of 14 days and a health education program to be carried out at home where recommendations for the care of health sequelae are included.

### 2.4. Outcome Measures

The initial assessment includes a self-made clinical interview for anamnesis conducted via videoconference.

#### 2.4.1. Affiliation Data and Socio-Demographic Questionnaire

Including age, gender, location and other socio-demographic variables.

#### 2.4.2. Main Explanatory Variable

##### Functional Capacity: For the Evaluation of the Functional Capacity and Fragility of the Patients the Following Tests Will Be Performed

Firstly, the “sit-to-stand test in 1 min” (1-min sit-to-stand, STS). This assessment of functional capacity in patients with respiratory impairment is an excellent alternative to the 6-min walk test (6MWT) which has certain limitations with regard to the space requirements for its execution. The 1-min STS test is a reliable, valid, and responsive test for measuring functional exercise capacity in patients with airway impairment and elicited a physiological response comparable to that of the 6MWT [37]. To carry out this test, the standardized protocol described by Crook and collaborators [37] will be used.

**Figure 2:**
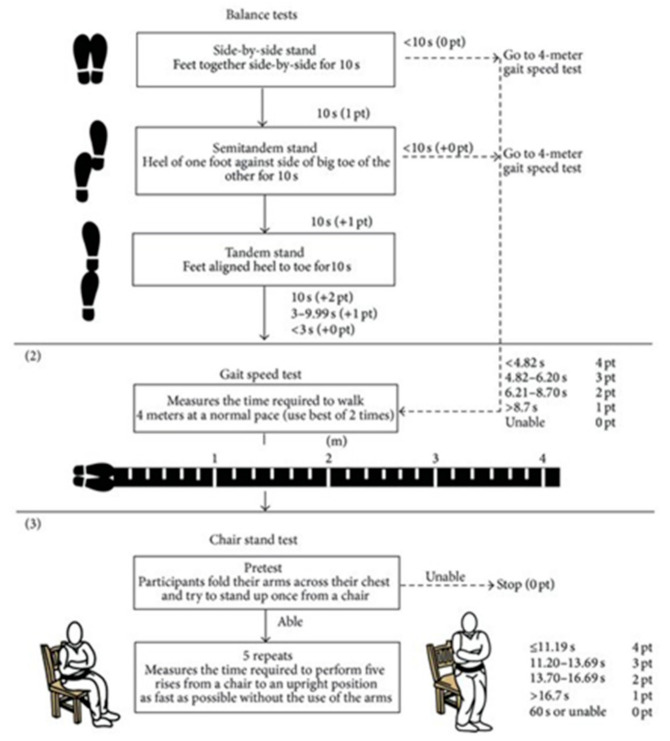
Short performance physical battery test (SPPB) based on Riskowski JL et al.

Secondly, the short performance physical battery test (SPPB). This is a widely used and validated test battery with high internal consistency, which evaluates three points: walking speed with the 4-m walk test; strength and resistance of the lower limbs by counting the time required to perform 5 squats (sit-to-stand test), and balance by standing with feet together, in tandem and semi-tandem. Each item is evaluated with a score from 0 to 4. The maximum final score is 12. An outline and protocol of the test execution is presented at Figure 2 [35].

##### Quality of Life

The SF-12 and EuroQoL-5d questionnaires will be used for the assessment of health-related quality of life.

The SF-12 questionnaire is the short version of the SF-36, presenting good correlation and being considered an adequate instrument of choice to measure the general health status of the population [38]. The SF-12 questionnaire assesses eight dimensions of health-related quality of life: physical function, physical role, body pain, general health, vitality, social function, emotional role and mental health [39]. The EuroQoL-5D assessment questionnaire (EQ-5D) contains a descriptive system of one’s health status measured in five dimensions (mobility, self-care, activities of daily living, pain and anxiety/depression). The EQ-5D has a visual analogue scale (VAS) that assesses the state of health “today” with scores ranging from 0 (worst imaginable health) to 100 (best imaginable health). With the EQ-5D questionnaire, information is obtained on the level of the problem (no problem, some/moderate problem or severe problem [39].

#### 2.4.3. Secondary Explanatory Variable 

##### Satisfaction and Usability

The acceptance and usability of telemedicine applications is a prerequisite for identifying the potential clinical benefits of this technology. It is therefore important to complement this research with tools that examine patient satisfaction and perception [40]. An adaptation of the Telemedicine Satisfaction and Usefulness Questionnaire (TSUQ), whose psychometric analysis supports construct validity and internal consistency reliability and which is available in English and Spanish will be used [40]. The instrument used showed high reliability (Cronbach’s alpha 0.8) and evidence of validity with respect to perception in telemedicine [41]. Includes 17 questions that are assessed with a 5-point Likert subjective scale (1 totally disagree and 5 completely agree). The individual obtains scores from 17 to 85. The higher the score, the better the perception of the telerehabilitation intervention. Therefore each of the scores of the 17 variables indicated and finally the total score of the Test will be analyzed [40,41].

##### Analysis of the Cost-Effectiveness

To assess the cost-effectiveness of the telerehabilitation intervention, the international guidelines for conducting cost analyses in randomized clinical trials will be followed [42]. Such an economic analysis is based on the health sector perspective, which means that only costs corresponding to health interventions will be considered, and not related costs to the patient. Therefore, only costs related to the provision of health services will be taken into account [43].

Costs are divided into two categories of variables: firstly, costs related to clinical aspects (direct costs) and secondly, costs related to the use of the technology (indirect costs).

Direct Costs: Numerical variable calculated on the basis of the number of hours of intervention and the cost of the Physiotherapist according to the hourly wage in force in the health system in the centers where the research is carried out. It will be calculated based on the recording of the number of sessions in both groups.

Indirect Costs: Numerical variable calculated based on the cost of using the technology for the telerehabilitation platform.

### 2.5. Data Collection Procedure and Statistical Analysis

Once the study subjects have been informed, data will be collected for statistical analysis. The procedure to register different outcomes will be performed at baseline T.0 (Pre), at 4 weeks after the start of the intervention T.1 (Post1), and 8 weeks when the intervention ends T.2 (Post). Schedule of enrollment, interventions, and assessments is shown in Table 1. Initial evaluation (Pre) and measures at four and eight weeks (Post 1 and Post 2) will be carried out by an evaluator at the headquarters designated for this purpose, facilitating the access of the patient and after confirmation of the appointment by the research secretary or by means of telecommunication tools that allow the realization of the different measuring instruments.

The data will be added to the database created for this purpose and managed by the principal researcher using exportable data tables for statistical analysis. Each of the participating subjects will be assigned a study participation number, and the results of each of the variables analyzed will be collected. The principal investigator will be in charge of the anonymization of the data, so that the data will be recorded by eliminating the link with the identifiable person.

#### Statistical Analysis

The results of the research will be presented as a summary of the outcome measures, together with the estimated effect size and its precision. The statistical analysis will be performed according to the intention-to-treat principle using SPSS software.

The filiation data of the participating subjects will be presented by means of frequency tables and histograms. A descriptive statistical analysis of the different variables will be carried out with frequency tables, bars and sectors in order to have as much information as possible for exploration and analysis. The homogeneity of the sample will be analyzed and normality tests will be performed.

The results are evaluated by comparing the differences between the groups with mixed linear model and T-test statistics to test the hypothesis that the means of two groups are or are not significantly different from each other.

## 3. Discussion

The development of this study could facilitate the creation of future clinical guidelines on the use of physical therapy through telerehabilitation platforms in the public health field.

The need to treat these patients in the short, medium and long term after discharge from hospital is an unavoidable commitment for the society that must assess the socio-economic impact and persevere in the search for effective interventions. This situation of world crisis is presented as an opportunity to continue developing professionally and scientifically, to implement the advantages offered by new technologies in the field of telerehabilitation, as well as to seek opportunities to solve the possible shortcomings of the Health Systems.

The current questions about the effects caused by COVID-19 should not limit us to act—on the contrary, it should stimulate the search for solutions that can bring pulmonary rehabilitation closer to patients who need it after discharge from hospital. In this sense, telerehabilitation is seen as a tool that could provide an efficient and cost-effective response to the health problems identified [12]. Physiotherapy will play a fundamental role, not only in the intensive care units and in the hospitalized patients, but it will also be a cornerstone in the interdisciplinary health team for the recovery of the sequelae which should lead to an improvement in the quality of life of patients with COVID-19 [12].

Telemedicine has the promise of improving quality, increasing patient access and reducing health care costs [44]. Studies have shown that telerehabilitation is effective in improving clinical outcomes in various pathologies suggesting that the increased intensity provided by telerehabilitation is a promising option to be offered to patients [27]. Recent research concludes that telerehabilitation appears to be a viable option for selected hospitalized patients with COVID-19 and may be a safe way of delivering inpatient rehabilitation to isolated or at-risk populations [45]. Although a recent systematic review indicates that telerehabilitation may be safe and feasible and may lead to reduced face-to-face rehabilitation therapy; in addition, remote rehabilitation assessment should be considered during the COVID-19 pandemic [46]. Another important fact is that the reported number of adverse events was low, and most studies reported that the average session adherence rate was >70% [46].

Unlike other studies that require software implementations in specific devices, our intervention generates few obstacles since it is available in any device that allows internet connection and that patients usually have (personal computer, laptop, tablet, smartphone) allowing access from any location and different devices. This contrasts with other studies that require a highly complex technological platform, software installation and multidirectional cameras for controlled clinical control connecting the healthcare provider and the patient [47]. As the patient uses his own technological devices, the rapid implementation process is guaranteed. This, however, may lead to a selection bias for those patients without access to technology. Technical problems (disconnection, device failures) and technological difficulties may arise in connection with the use of technology. However, staff members will be available to provide technical support to patients by phone or email, without the need to visit the patient at home to install or check any hardware.

The lack of improvement and positive evolution of the patient, as well as the appearance of a low level of adherence was considered as possible adverse events. Also is considered as an adverse event, the performance of movements with excessive workload that cause muscle overload.

Patients will be informed of the importance of warning the healthcare professional of any incident or setback in their recovery, as well as their right to withdraw from participation in the research at any time.

## 4. Conclusions

This research should provide knowledge about the possibility of implementing TR programs in patients with COVID-19 after discharge from hospital, identifying health resources and allocated costs that will allow the definition of new intervention policies in this group of patients.

## Figures and Tables

**Table 1:** Schedule of enrolment, interventions, and assessments.

	Enrolment	Allocation	Post Allocation		Close out
Time Point	−T 1	T 0 [Baseline]	T.1 [4 Weeks]	T.2 [8 Weeks]	
ENROLMENT:					
Eligibility screen	X				
Informed consent	X				
Advance info	X				
Allocation		X			
INTERVENTION:					
TR Group		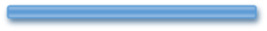			
Control Group		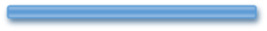			
ASSESMENT:					
Baseline Variables	X				
Initial Assesment		X			
STS Test		X	X	X	X
SPPB test.		X	X	X	X
SF-12 and EQ-5D		X	X	X	X
TSUQ			X	X	X
Direct Costs			X	X	X
Data Collection		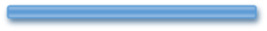			
Statistical Análisis					X

## Data Availability

The Researcher declares that he follows the protocols of his work centre regarding the publication of data in accordance with the provisions of Organic Law 15/1999, of 13 December, on the Protection of Personal Data (LOPD), and that the data will be incorporated into a file for the purpose of carrying out this research project. Participating subjects will be informed of the possibility of exercising their rights of access, rectification, cancellation and opposition of their data at the e-mail address provided by the principal investigator.

## Abbreviations

SARS-CoV-2: Severe Acute Respiratory Syndrome Coronavirus. COVID19: Coronavirus Disease 2019. TR: Telerehabilitation. SQUIRE: Standards for Quality Improvement and Excellence in Reporting. SPIRIT: Standard Protocol Items Recommendations for Interventional Trials. IC: confidence index. CG: Control Group. STS: sit-to-stand test in 1 min. 6MWT: 6-min walk test. SPPB: short performance physical battery test. SF-12: Short Form Health Survey 12. EuroQoL-5d: European Quality of Life-5 Dimensions survey. SF-36: Short Form Health Survey 36, VAS: visual analogue scale. TSUQ: Telemedicine Satisfaction and Usefulness Questionnaire. CONSORT: Consolidated Standards of Reporting Trials
